# The Adsorption of Ochratoxin A by *Lactobacillus* Species

**DOI:** 10.3390/toxins6092826

**Published:** 2014-09-22

**Authors:** Małgorzata Piotrowska

**Affiliations:** Institute of Fermentation Technology and Microbiology, Faculty of Biotechnology and Food Science, Lodz University of Technology, Wólczańska 171/173, Łódź 90-924, Poland; E-Mail: malgorzata.piotrowska@p.lodz.pl; Tel.: +48-42-631-34-70; Fax: +48-42-636-59-76

**Keywords:** ochratoxin A, *Lactobacillus*, adsorption, hydrophobicity, cell wall

## Abstract

The objective of this study was to examine ochratoxin A (OTA) binding by three lactic acid bacteria (LAB) species: *Lactobacillus plantarum*, *L. brevis*, and *L. sanfranciscensis*. Experiments were conducted using MRS medium and PBS buffer contaminated with 1000 ng/mL OTA and inoculated with live or thermally inactivated bacterial biomass at a concentration of 1 or 5 mg dry weight/mL. It was found that, depending on the strain and biomass density, live bacterial cells reduced OTA content by 16.9% to 35% in MRS medium and by 14.8% to 26.4% in PBS after 24 h of contact. OTA binding was higher in the case of thermally inactivated bacterial biomass (46.2% to 59.8%). The process is very rapid: OTA was removed from PBS as early as after 30 min of contact. The binding of the toxin by cells was partially reversible under the treatment by water and 1 M HCl. The results show that OTA is adsorbed to the surface structures of the cell wall, which is promoted not only by the hydrophobic properties of the cell wall, but also by electron donor-acceptor and Lewis acid-base interactions.

## 1. Introduction

Ochratoxin A consists of L-phenylalanine linked to a coumarin derivative by a peptide bond involving the amino group. It is produced by molds of the genera *Aspergillus* and *Penicillium* [1]. In the Polish climate, the main ochratoxin A (OTA) producers are *Penicillium verrucosum*, *P. nordicum*, *P. aurantiogriseum*, as well as *Aspergillus* section *Nigri* [1,2,3,4]. The amount of toxin produced depends not only on the type of medium and mold growth conditions, but also on the mold species and strain [5]. In humans and animals, this toxin often leads to diseases of the excretory system. OTA has been shown to have nephrotoxic and nephrocarcinogenic, immunosuppressive, neurotoxic, and teratogenic activity. It causes nephropathy involving renal tubular atrophy, interstitial fibrosis of renal cortex, and urinary tract tumors [6,7].

Ochratoxin A is a contaminant of plant- and animal-based food and food products. As a result of its thermal stability, it may also be detected in heat-processed food [8]. 

EU regulations set forth limits on ochratoxin contamination for cereal products, coffee, wine, dried fruits, spices, and licorice [9]. A Polish study conducted in the years 2004–2006 revealed the presence of OTA in 42 out of 107 examined food samples, but in most cases the contamination levels were low and did not exceed the official limits [10]. The large scale of mycotoxin contamination is attested to by the high number of alert and information notifications issued under the Rapid Alert System for Food and Feed (RASFF), which is a platform for exchanging information about foods and feeds that may pose a hazard to the consumers. In the years 2008–2012, 148 RASFF notifications were devoted to food products contaminated with ochratoxin A; these mostly included dried grapes, cereals, cereal products, and spices [11,12].

Due to the harmful effects of mycotoxins on human and animal health and the economic losses caused by food and feed contamination, it is necessary to minimize the risk of exposure to these compounds, mostly through preventive measures, such as appropriate treatment of plant materials during cultivation, harvest, and storage [13]. However, such measures are not always effective as mycotoxins are often formed during the storage and distribution of the final products as a result of excessive dampness conducive to mold growth. Treatments intended to reduce mycotoxin levels, such as thermal treatment or the addition of oxidants or ammonia, are used in the animal feed industry, but cannot be applied to crops and foods intended for humans. Physical and chemical decontamination methods have been reviewed in many papers [14,15,16,17], but due to their limitations other, safer techniques should be sought, including microorganism-based biological methods. Many studies have shown that some microorganisms can remove toxins from the environment; these include the bacteria *Lactobacillus acidophilus*, *L. plantarum*, *Acinetobacter calcoaceticus*, *Bifidobacterium animalis*, and *Oenococcus oeni*; the molds *Aspergillus niger*, *A. carbonarius*, and *A. fumigatus*; and the yeasts *Saccharomyces cerevisiae*, *Kloeckera apiculata*, and *Kluyveromyces marxianus* [18,19,20,21,22,23,24,25]. This issue has also been the subject of a number of review papers [26,27,28,29]. Among the microorganisms exhibiting decontamination properties of particular interest are lactic acid bacteria, as they are safe, have beneficial effects on human health, and bind mutagens from food and the environment [30,31]. Furthermore, they are often used in fermentation processes, e.g., in the baking industry, where flour and other ingredients may be contaminated with OTA. Previous research has shown that all 29 strains of *Lactobacillus* and *Lactococcus* bacteria reduce OTA content in growth media. The greatest toxin elimination ability has been found for the enteric bacteria *Lactobacillus rhamnosus* and *L. acidophilus* as well as for the plant-associated bacteria, such as *L. plantarum*, *L. brevis*, and *L. sanfranciscensis* [22]. The objective of the presented study is to elucidate the process of ochratoxin A removal by three *Lactobacillus* strains and to determine the mechanism responsible for this process.

## 2. Results and Discussion

It should be noted that mycotoxins exhibit antibiotic properties, inhibiting microbial growth. Therefore, in researching decontamination processes, one should select those bacterial species that are not susceptible to the presence of ochratoxin A in the environment. As was shown in a previous screening study, lactic acid bacteria are generally not very susceptible to OTA, but there exist some differences between species and strains [22].

The experiments were conducted using three plant-associated lactic acid bacteria (LAB) strains: *Lactobacillus plantarum*, *L. brevis*, and *L. sanfranciscensis*. They were selected out of six strains belonging to the same species based on comparison of biomass increase in cultures contaminated with 1000 ng/mL OTA *versus* control media (Table 1).

**Table 1:** The effect of ochratoxin A on biomass yield of lactic acid bacteria.

Strain	Log (*N*/*N*_0_) after 24 h of incubation	
	MRS + OTA	Control MRS
*Lactobacillus plantarum* LOCK 0861	4.02 ± 0.12 ^a,A^	4.65 ± 0.09 ^a,B^
*Lactobacillus plantarum* LOCK 0862	4.75 ± 0.14 ^c,A^	4.83 ± 0.16 ^a,A^
*Lactobacillus plantarum* LOCK 0863	4.15 ± 0.20 ^a,A^	4.78 ± 0.13 ^a,B^
*Lactobacillus brevis* LOCK 0845	3.95 ± 0.11 ^a,A^	4.02 ± 0.23 ^b,B^
*Lactobacillus sanfranciscensis* LOCK 0866	4.56 ± 0.16 ^c,A^	4.76 ± 0.09 ^a,A^
*Lactobacillus sanfranciscensis* LOCK 0867	3.76 ± 0.03 ^b,A^	4.23 ± 0.18 ^b,B^

Note: *N*—the number of bacteria after 24 h (CFU/mL); *N*_0_—the number of bacteria in *t* = 0 h (CFU/mL); MRS—MRS medium; the results are given as mean values from three experiments ± standard deviation; statistically significant differences were found between data designated with the different letters (A,B) in a given row (one-way ANOVA, *p* < 0.05); statistically significant differences were found between data designated with the different letters (a–c) in a given column (one-way ANOVA, *p* < 0.05).

After 24 h of incubation, the number of cells in MRS medium increased by approximately 4 log units as compared to the inoculum. However, in the case of some strains OTA had a negative influence on biomass yield. Differences in biomass yield between the OTA-contaminated medium and the control medium were statistically significant for the strains *L. plantarum* LOCK 0861 and LOCK 0863, *L. brevis* LOCK 0845, and *L. sanfranciscensis* LOCK 0867 (*p* < 0.05), but did not exceed 1 log(*N*/*N*_0_) unit. These results are similar to those reported in the literature, where OTA, at a much higher dose (20 µg/mL), did not inhibit the growth of *L. plantarum* or *L. casei* [32]. Based on the obtained results, for the further study *Lactobacillus plantarum* LOCK 0862 and *Lactobacillus sanfranciscensis* LOCK 0866 strains were chosen, and additionally *Lactobacillus brevis* LOCK 0845.

Experiments concerning OTA removal from culture media were conducted using two different bacterial biomass concentrations (1 and 5 mg dw/mL). The percentage reduction of OTA in MRS medium differed depending on biomass density. At the initial concentration of 1 mg dry weight (dw)/mL, the bacteria removed 16.91% to 21.23% of OTA, while at a fivefold higher concentration, they removed 20.53% to 35.01% of OTA (Table 2). Furthermore, some interstrain differences were noted (*p* < 0.05). During 24 h culture in MRS medium, *L. plantarum* and *L. sanfranciscensis* removed more than 30% of the initial toxin content (at an inoculum concentration of 5 mg dw/mL). *Lactobacillus brevis* was found to be the least effective, as it reduced the initial toxin content by only 20.5%. This may have resulted from growth differences between the species during 24 h incubation. As can be seen in Table 1, *Lactobacillus brevis* was the only one of the selected species whose growth was inhibited by OTA. The results obtained for MRS medium are consistent with literature data. According to El-Nezami and co-workers [19] and Fusch and co-workers [21], for toxin elimination to be effective, the concentration of microbial cells must be high, 10^9^ CFU/mL or more. This is corroborated by the fact that the amount of removed OTA was greater at a higher bacterial biomass concentration. 

Experiments conducted in PBS, which does not contain any nutrients for microorganisms and thus prevents their growth, showed smaller differences than those found for MRS medium. Additionally, here, a better decontamination effect was observed at a bacterial biomass concentration of 5 mg dw/mL (*p* < 0.05). The percentage reduction of OTA relative to its initial content was similar to that observed for MRS medium and amounted to 14.8%–16.1% and 22.5%–26.4% at a lactic acid bacterial biomass concentration of 1 and 5 mg/mL, respectively (Table 2). For comparison purposes, experiments were also conducted on the gram-negative bacteria *Escherichia coli*. At a biomass concentration of 5 mg dw/mL, OTA content was reduced by only 2.66%, which is not much different from the control sample (without biomass). This is in accordance with the literature data, as *E. coli* has not been found to eliminate mycotoxins. In addition, in the study by El-Nezami and co-workers [19] *E. coli* did not reveal decontamination properties regarding aflatoxin B_1_, which the authors attributed to the structure of its cell wall, that is, a low content of peptidoglycan and the presence of lipopolysaccharides. The obtained OTA elimination results for the buffer solution were similar to those reported by Fusch and co-workers [21] (nearly 40% elimination by *Lactobacillus plantarum* at the same initial inoculum concentration). 

**Table 2:** The effect of bacterial biomass amount and physiological state on ochratoxin A removal.

Strain	Biomass density (mg s.m./mL)	Toxin reduction % after 24 h			
		MRS	PBS buffer		
			LB	DB	S *
*L. plantarum* LOCK 0862	1	21.23 ± 1.56 ^a,A^	15.35 ± 0.46 ^a^^,b,B^	48.01 ± 0.95 ^a,C^	3.91 ± 0.20 ^a,D^
	5	35.01 ± 1.25 ^c,A^	26.42 ± 0.45 ^c,B^	56.16 ± 0.44 ^b,C^	ns
*L. brevis* LOCK 0845	1	14.64 ± 0.61 ^b,A^	16.12 ± 0.70 ^a,A^	46.29 ± 0.78 ^c,B^	5.20 ± 0.36 ^b,C^
	5	20.53 ± 0.87 ^a,A^	24.23 ± 0.36 ^d,B^	59.82 ± 0.56 ^d,C^	ns
*L. sanfranciscensis* LOCK 0866	1	16.91 ± 0.26 ^d,A^	14.80 ± 0.46 ^b,B^	52.72 ± 0.70 ^e,C^	5.62 ± 0.20 ^b,D^
	5	32.00 ± 0.79 ^e,A^	22.52 ± 0.35 ^e,B^	56.40 ± 0.78 ^b,C^	ns
*Escherichia coli* ATCC 10536	5	ns	2.66 ± 0.61 ^f^	ns	ns

Note: LB—live biomass; DB—dead biomass; S—spheroplasts; ns—not studied; the results are mean values from three experiments ± standard deviation; different letters (a–f) in columns designate statistically significant differences between strains and biomass concentrations (*p* < 0.05); different letters (A–D) in rows designate statistically significant differences between media and physiological state of bacteria (one-way ANOVA, *p* < 0.05); * spheroplasting effectiveness—approximately 90%.

The fact that LAB decreased the amount of OTA not only in a microbiological medium, but also in PBS shows that decontamination does not require cells in an active growth phase. In a medium in which OTA is the only source of carbon, toxin elimination may proceed in two ways—by biodegradation or by binding to cells. To confirm one or the other mechanism, experiments involving thermally inactivated bacterial biomass were conducted. Thermally inactivated biomass led to higher decontamination effectiveness than live cells: dead bacterial cells at a concentration of 1 mg dw/mL bound 46.29% to 52.72% of the initial toxin content, while the results for live biomass were 14.80% to 16.12% under the same conditions. In the case of the *Lactobacillus brevis* strain, dead bacterial cells at a concentration of 5 mg dw/mL resulted in an OTA reduction of 59.82%, which is almost twice more than for life cells. The same trend was observed during the incubation of the other two species. However, no direct relationship was found between biomass concentration and process effectiveness (e.g., a five-fold increase in biomass content did not cause a corresponding increase in process effectiveness (Table 2)). 

The obtained results show that OTA is removed from the media also in the absence of metabolically active cells, which indicates that the toxin is adsorbed to the cells, and the more cells there are in the suspension, the greater the amount of OTA that can be eliminated. Thermally inactivated (dead) cells exhibited decontamination properties that were several times higher than those found for live biomass at the same concentration. Toxin-to-cell binding may be of physicochemical nature. Toxin adsorption by dead biomass has been reported for yeasts eliminating OTA from grape must and for LAB binding aflatoxin B_1_ [19,23,33,34]. In contrast, Fusch and co-workers [21] showed that thermally inactivated LAB cells removed very little OTA from the medium (less than 11%), as opposed to metabolically active cells, which eliminated as much as 96% of the toxin.

Higher OTA adsorption by dead rather than live cells may be explained by changes occurring in the bacterial cell wall induced by high temperature, that is, protein denaturation and pore generation leading to increased permeability of the external layers of the cell wall (this in turn results in a greater number of active sites responsible for the sorption of different compounds) [34]. The adsorption of bacteria by dead biomass is highly beneficial and may be used in practice, as this decontamination method facilitates the preservation of the organoleptic properties of the products, while bacterial cells may be used as dietary supplements preventing the absorption of toxins in the human gastrointestinal tract. According to Tuomola and co-workers [35], high temperatures decrease the adhesion of bacteria to the intestinal mucosa, thanks to which these microorganisms may be eliminated from the body together with the toxins adsorbed on them. Consequently, the cells containing toxins are not likely to remain in the intestines, from where they could be absorbed. It has been suggested that adhesion to the mucosa decreases with the degree of denaturation of the proteins responsible for that process, while the hydrocarbons binding OTA are not degraded [34,36,37].

**Figure 1:**
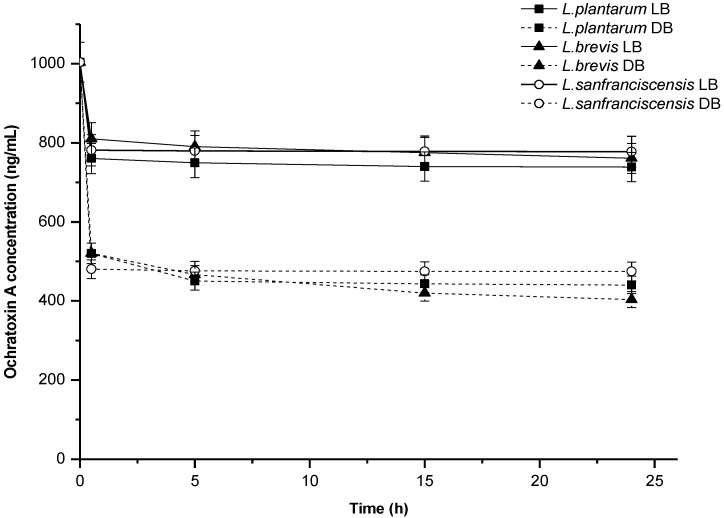
Dynamics of ochratoxin A removal by lactic acid bacteria in PBS; biomass concentration—5 mg dw/mL, LB—live biomass, DB—dead biomass.

In terms of the dynamics of OTA removal in PBS, it was found that as early as after 30 min of contact between live bacterial biomass and the toxin only 760 to 810 ng/mL OTA remained in the buffer. This means that more than 80% of total OTA removed throughout the 24 h experiment was adsorbed during the first half hour. The process of OTA elimination by dead biomass was equally rapid. Most OTA was adsorbed during the first two hours of incubation, and no major changes were observed until the end of the experiment, that is, 24 h (Figure 1). These results are consistent with the literature data [19].

Experiments with spheroplasts were conducted to elucidate the role of the bacterial cell wall. It was found that cells, which had their cell wall partially removed, bound OTA very poorly (from 3.9% to 5.6%), which proves that the presence of the cell wall is indispensable for the adsorption process to occur (Table 2). According to the literature, the key role in the binding of mycotoxins by bacterial biomass is played by cell wall components, such as peptidoglycan and polysaccharides, as well as teichoic and lipoteichoic acids [38,39]. The factor responsible for toxin removal may be the hydrophobic nature of the bacterial cell wall [34,37].

Prior to a future application of this method for toxin inactivation, the stability of the toxin-cell wall complex should also be studied. If the binding is weak, mycotoxins will be released due to continuous washing of the bacteria in the gastrointestinal tract. Therefore, in the next step of the study, the bacterial biomass was washed with distilled water and hydrochloric acid, and the amount of released OTA was determined in the supernatant following centrifugation. The results are presented in Table 3.

The binding of the toxin to the biomass is not very stable, as some OTA was released under the influence of washing with water alone. Triple washing of live cells with water led to desorption of 71.88 to 78.09 ng/mL OTA. Even more OTA was released by thermally inactivated dead cells (159.84 to 183.46 ng/mL). Irrespective of the physiological state of the cells, this amounted to approximately. 30% of previously bound OTA. The action of hydrochloric acid led to releasing OTA amounts similar to those desorbed as a result of washing with water. This means that the toxin will not be excessively released in the acidic environment of the stomach, which would be highly undesirable. The reversibility of aflatoxin binding to LAB has been also shown by Haskard and co-workers [37]. Del Prete and co-workers [40] reported that LAB isolated from wine released from 31% to 57% of the OTA adsorbed by the cells as a result of washing the biomass with a buffer solution. In the study of Niderkorn and co-workers [39], the treatment of LAB cells with 1 M HCl led to increased binding of fumonisin by *Streptococcus* cells, while releasing previously bound toxins.

**Table 3:** Ochratoxin A desorption from cells of lactic acid bacteria.

Wash medium	Wash no.	Strain					
		*L. plantarum* LOCK 0862		*L. brevis* LOCK 0845		*L. sanfranciscensis* LOCK 0866	
		LB	DB	LB	DB	LB	DB
Water	I	*50.31 ± 0.22 ^a^	121.2 ± 0.33 ^c^	49.53 ± 0.66 ^a^	115.71 ± 0.84 ^d^	51.20 ± 0.22 ^b^	119.84 ± 1.48 ^c^
	II	21.12 ± 0.73 ^a^	56.05 ± 0.38 ^d^	18.71 ± 0.44 ^b^	38.92 ± 0.34 ^e^	23.65 ± 0.47 ^c^	46.47 ± 1.44 ^f^
	III	4.21 ± 0.13 ^a^	6.26 ± 0.25 ^e^	3.64 ± 0.22 ^b^	5.21 ± 0.22 ^d^	3.24 ± 0.10 ^c^	4.91 ± 0.30 ^d^
HCl	I	60.36 ± 1.28 ^a^	139.51 ± 0.54 ^d^	56.20 ± 0.70 ^b^	125.32 ± 0.58 ^e^	54.72 ± 0.26 ^c^	128.27 ± 0.73 ^f^
	II	18.77 ± 0.24 ^a^	28.42 ± 0.61 ^d^	10.66 ± 0.71 ^b^	28.46 ± 0.53 ^d^	16.81 ± 0.32 ^c^	25.61 ± 0.51 ^e^
	III	5.34 ± 0.25 ^b,d^	7.27 ± 0.24 ^c^	4.65 ± 0.38 ^a,b^	5.87 ± 0.17 ^e^	4.31 ± 0.32 ^a^	5.87 ± 0.56 ^d,e^

* OTA content in the supernatant (ng/mL); LB—live biomass; DB—dead (thermally inactivated) biomass. Different letters (a–f) in rows designate statistically significant differences between strains and physiological state of bacteria (one-way ANOVA, *p* < 0.05). The differences between columns are not statistically significant (*p* > 0.05).

According to literature data, the adsorption of mycotoxins to the cell wall of LAB is attributable to their surface properties, and mainly to their hydrophobicity [34]. According to Chae and co-workers [41], cells are strongly hydrophobic if their percentage adhesion to hexadecane exceeds 55%, moderately hydrophobic for 30%–54% adhesion, moderately hydrophilic for 10%–29% adhesion, and strongly hydrophilic for <10% adhesion. In the present study, based on hexane adhesion experiments, the surface of live LAB cells was found to be strongly hydrophilic, while that of *Escherichia coli* cells was moderately hydrophilic (Table 4). This is consistent with Boonaert and Rouxhet [42], who reported that the surface of *Lactobacillus helveticus* and *Lactococcus lactis* is hydrophilic, irrespective of their growth phase. The authors attributed this fact to the low content of carbohydrates in the outer layers of the cell wall. In addition, *Lactobacillus casei*, *L. paracasei*, and *L. rhamnosus* cells have been found to be hydrophilic [43]. Following thermal treatment, the surface of all the *Lactobacillus* bacteria examined in this study became hydrophobic as hexadecane adhesion exceeded 35% (Table 4). Thermally inactivated cells were more effective in terms of toxin removal, which indicates that the hydrophobic surface is responsible for that process. This is in accordance with the literature data [37]. The fact that OTA was bound also by live LAB, despite the hydrophilic nature of their surface, is probably attributable to the presence of so-called hydrophobic pockets on the surface [34]. While the surface of *E. coli* revealed similar characteristics, it was not found to bind toxins. This shows that toxin adsorption is also affected by other factors, such as the chemical composition of the cell wall, which contains more lipopolysaccharides in Gram-negative bacteria. The low adhesion to ethyl acetate, which is a strongly basic solvent (<15% for live cells and >20% for thermally inactivated cells) indicates that the cell surface is basic. In turn, the high adhesion of both live and dead LAB to chloroform (>35%) shows considerable affinity of LAB to this acidic solvent, which is an electron acceptor [43,44]. The OTA adsorption may be related to presence of the outer S-layer envelope attached to cell wall components. Many species of the genus *Lactobacillus,* including *L. brevis* possess a surface layer composed of proteins or glycoproteins with a higher number of positively charged residues than negatively charged residues [45,46]. Schut and co-workers [47] reported that the *Lactobacillus hilgardii* cells with *S*-layer can effectively bind Cu and Fe by negatively charged carboxyl and phosphorous residues on their surface. However, the cells without *S*-layer proteins, after treatment by proteinase K, adsorbed more Zn and Mn than cells with this compound [47]. According to Schut and co-workers [47] the *Lactobacillus plantarum* has no *S*-layers and has different behavior relating to the binding of metal ions. However, in the presented study, the significant differences between *L. brevis* and *L. plantarum* binding capacity were not observed, which suggested that, in the case of ochratoxin A, the role of *S*-layer protein compounds are not relevant.

According to the presented results, cells of lactic acid bacteria are strong electron donors and weak electron acceptors, which is supported by the hydrophilic nature of their surface. Therefore, it may be assumed that the binding of toxins by bacterial cells is also influenced by electron donor-acceptor and Lewis acid-base interactions. 

**Table 4:** Characterization of bacterial surface—adhesion to solvent (MATS method).

Solvent	Strain						
	*L. plantarum* LOCK 0862		*L. brevis* LOCK 0845		*L. sanfranciscensis* LOCK 0866		*E. coli* ATCC 10536
	LB	DB	LB	DB	LB	DB	LB
HD	*7.63 ± 0.35 ^a^	38.52 ± 0.05 ^b^	6.89 ± 0.09 ^c^	35.34 ± 0.56 ^d^	5.67 ± 0.06 ^e^	36.72 ± 0.58 ^f^	21.13 ± 0.09 ^g^
CH	59.30 ± 0.24 ^a^	42.91 ± 0.32 ^b^	53.45 ± 0.23 ^c^	45.21 ± 0.45 ^d^	51.21 ± 0.16 ^e^	47.82 ± 0.36 ^f^	8.78 ± 0.06 ^g^
EA	12.51 ± 0.05 ^a^	23.43 ± 0.41 ^b^	13.56 ± 0.38 ^c^	25.76 ± 0.08 ^d^	14.53 ± 0.23 ^e^	24.37 ± 0.41 ^f^	30.85 ± 0.28 ^g^

Note: * % of adhesion to solvents; HD—hexadecane; CH—chloroform; EA—ethyl acetate; LB—live biomass; DB—thermal inactivated biomass; different letters (a–g) in rows designate statistically significant differences between strains and physiological state of bacteria (one-way ANOVA, *p* < 0.05).

## 3. Experimental Section

### 3.1. Biological Material

The biological material consisted of LAB strains: *Lactobacillus plantarum* LOCK 0862, LOCK 0861, LOCK 0863, *Lactobacillus brevis* LOCK 0845, *Lactobacillus sanfranciscensis* LOCK 0866 and LOCK 0867, and *Escherichia coli* ATCC 10536. The strains were obtained from the Pure Culture Collection of the Institute of Fermentation Technology and Microbiology, Lodz University of Technology (LOCK) and the American Type Culture Collection (ATCC). LAB were stored frozen in MRS medium (Merck GmbH, Darmstadt, Germany) with 15% *v*/*v* glycerol, while the *E. coli* strain was stored on slants with TSA (Merck GmbH, Darmstadt, Germany). The strains were activated by subculturing in MRS or TSB liquid media (Merck GmbH, Darmstadt, Germany). Incubation was conducted at 30 °C for 24 h. Subsequently, the cultures were centrifuged (7000 × *g* for 10 min), the biomass was washed with water, suspended in sterile water, and used for further study. 

### 3.2. Chemicals

All chemicals and solvents were purchased from Sigma-Aldrich, St. Louis, MO, USA and were of analytical grade. Water from a Millipore Milli-Q system (Millipore, Bedford, MA, USA) was used for all solutions, dilutions, and HPLC mobile phase. Ochratoxin A was stored as a stock solution in absolute ethanol (HPLC grade) at −20 °C. The concentration of OTA in stock solution was 200,000 ng/mL. 

### 3.3. The Influence of Ochratoxin A on Bacterial Growth

A closed culture of LAB was conducted for 24 h in MRS medium contaminated with OTA and in a control medium without the toxin. The concentration of OTA in the medium was 1000 ng/mL, while the initial concentration of bacteria was approximately 10^4^ CFU/mL. The increase in the number of bacteria following 24 h of incubation at 30 °C was determined in MRS medium by the plate method and expressed as log(*N*/*N*_0_) (*N*—number of CFU/mL after 24 h; *N*_0_—number of CFU/mL at the beginning of incubation). 

### 3.4. Ochratoxin A Binding Assay

Ochratoxin A removal by LAB was studied in MRS medium and PBS buffer contaminated with OTA (Sigma-Aldrich, St. Louis, MO, USA). The initial OTA concentration was 1000 ng/mL. OTA content in the media was determined each time. Growth media were inoculated with a suspension of bacterial biomass so that its initial concentration in the media was 1 and 5 mg dry weight (dw)/mL. Suspension density was standardized with the spectrophotometric method (540 nm). Incubation was conducted under closed culture conditions at 30 °C for 24 h. The control sample consisted of the same mixture, but without OTA. For comparative purposes, experiments involving *Escherichia coli* were additionally conducted in PBS, using suspension with a microbial concentration of 5 mg dw/mL. 

At the beginning of incubation (*t* = 0), and after 30 min, 10 h, 15 h, and 24 h, samples were taken from the culture. The samples were centrifuged at 7000 × *g* for 10 min and the amount of residual OTA was determined in the supernatant. The reduction of OTA content, relative to the initial value, was calculated.

### 3.5. Ochratoxin A Binding by Dead LAB Biomass

LAB suspensions in sterile water were prepared and autoclaved at 121 °C under a pressure of 1 atm for 15 min. The effectiveness of thermal inactivation of the microorganisms was verified by plating on MRS media.

Toxin binding was studied in PBS medium according to the protocol given above. At the beginning of incubation (time 0), and at 16 h and 24 h following biomass centrifugation, the amount of residual OTA was determined in the supernatant. The reduction of OTA content relative to the initial value was calculated.

### 3.6. Ochratoxin A Binding by Bacterial Spheroplasts

In order to elucidate the role of the cell wall in toxin removal, experiments were conducted in PBS using bacterial spheroplasts obtained according to the procedure described by Tanaka and Ohmomo [48]. Bacterial strains were activated in MRS medium containing gelatin and glycine (1%). After 24 h, the culture was transferred to a fresh MRS medium with gelatin and glycine and was incubated at 4 °C for 24 h and at 37 °C for 3 h. Subsequently, the culture was centrifuged (7000 × *g*, 10 min), washed with water twice, and centrifuged again. The biomass was suspended in SA buffer containing raffinose (sodium acetate 0.615%, EDTA 0.1%, MgCl_2_ 0.254%, raffinose 29.72%, pH 6.2). A solution of lysozyme in SA buffer (6 mg/mL) was added, and then the suspension was incubated at 37 °C for 1 to 2 h. Protoplasting was monitored microscopically. Additionally, the suspension was plated on MRS medium without an osmotic stabilizer to determine the number of cells that did not undergo spheroplasting and calculate the percentage degree of spheroplasting relative to the initial cell count. The binding of OTA by bacterial spheroplasts in PBS was calculated following microscopic determination of spheroplast concentration (10^8^/mL). Subsequent steps were carried out in the manner described above. 

### 3.7. Stability of Toxin Binding to the Bacterial Biomass

The stability of toxin binding to the bacterial biomass was verified during the desorption process using water and HCL. Following experiments on the binding of OTA by live and dead bacterial biomass in PBS (5 mg dw/mL), the biomass was centrifuged, and then vortexed three times for 5 min with distilled water or 1 M HCl. Each time the mixture was centrifuged and supernatant fractions were collected (fractions I, II, and III). The amount of OTA in supernatant was determined. 

### 3.8. Bacterial Cells Surface Properties

Cell surface properties were determined according to the protocol proposed by Pelletier and co-workers [43] by the microbial adhesion to solvents (MATS) method (for live and thermally inactivated *Lactobacillus* strains and for live *Escherichia coli* bacteria (for comparative purposes). First, a 24 h culture of bacteria in a liquid medium was centrifuged (5000 × *g*, 10 min), washed with water twice, and then resuspended to an optical density of 0.4 at 400 nm (*A*_0_) in 0.1 M KNO_3_. A similar suspension was prepared using thermally inactivated cells. In the next step, 0.2 mL of solvent was added to 1.2 mL of cell suspension. After 10 min preincubation at ambient temperature, the mixture was vortexed for 2 min. After 15 min the aqueous phase was removed and its absorption was measured at 400 nm (*A*_1_). Percentage of bacterial adhesion to solvents was calculated as (1 − *A*_1_/*A*_0_) × 100. Three different solvents were used: hexadecane (non-polar), chloroform (acidic monopolar); and ethyl acetate (basic monopolar). Hexadecane adhesion testing shows whether the cell surface is hydrophobic or hydrophilic. The MATS values obtained for chloroform and ethyl acetate determine the donor/basic and acceptor/acidic properties of cells surface, respectively, as well as the presence of Lewis acid-base interactions [44].

### 3.9. Ochratoxin A Determination

Extraction and sample clean-up were performed using an OchraStar^®^ immunoaffinity column (Romer Labs^®^ Diagnostic GmbH, Tulln, Austria) according to the manufacturer’s instructions. Ochratoxin A was determined by HPLC using a Finnigan™ Surveyor Plus™ chromatograph (Thermo Separation Products, Riviera Beach, FL, USA) with an Ace 5 µm C18 column (250 mm × 4.6 mm) and an Ace 5 C18 guard column (Advanced Chromatography Technologies, Aberdeen, UK), loop 50 µL, flow rate 1 mL/min, ambient temperature, mobile phase—water:acetonitrile:acetic acid (99:99:2 *v*/*v*/*v*), fluorescence detection (λ_excitation_ = 330 nm, λ_emission_ = 460 nm). 

### 3.10. Statistical Analysis

The presented results are mean values from three independent experiments. Statistical analysis (means, standard variation) and analysis of variance (one-way ANOVA) were conducted using ORIGIN, v.6.1, Microcal, Northampton, MA, USA, 2000 and Statistica, v.10.0, StatSoft, Inc., Tulsa, OK, USA, 2011 software.

## 4. Conclusions

The aim of the presented study was to elucidate the process of ochratoxin A removal by three *Lactobacillus* strains and to determine the mechanism responsible for this process. It was found that OTA is also removed from the media in the absence of metabolically active cells, which indicates that the toxin is adsorbed to the cells. Thermally inactivated (dead) cells exhibited decontamination properties that were several times higher than those found for live biomass at the same concentration. The binding of the toxin to the biomass is not very stable, as some OTA was released under the influence of washing with water or acid. Toxin-to-cell binding may be of physicochemical nature. OTA is adsorbed to the surface structures of the cell wall, which is promoted, not only by the hydrophobic properties of the cell wall, but also by electron donor-acceptor and Lewis acid-base interactions. 

The results showed that the application of lactic acid bacteria strains in different biotechnological processes involving raw material contaminated with OTA might reduce the toxin contamination, as well as the health risk related to human exposure to this toxin.
